# Ad26 vaccine protects against SARS-CoV-2 severe clinical disease in hamsters

**DOI:** 10.1038/s41591-020-1070-6

**Published:** 2020-09-03

**Authors:** Lisa H. Tostanoski, Frank Wegmann, Amanda J. Martinot, Carolin Loos, Katherine McMahan, Noe B. Mercado, Jingyou Yu, Chi N. Chan, Stephen Bondoc, Carly E. Starke, Michael Nekorchuk, Kathleen Busman-Sahay, Cesar Piedra-Mora, Linda M. Wrijil, Sarah Ducat, Jerome Custers, Caroline Atyeo, Stephanie Fischinger, John S. Burke, Jared Feldman, Blake M. Hauser, Timothy M. Caradonna, Esther A. Bondzie, Gabriel Dagotto, Makda S. Gebre, Catherine Jacob-Dolan, Zijin Lin, Shant H. Mahrokhian, Felix Nampanya, Ramya Nityanandam, Laurent Pessaint, Maciel Porto, Vaneesha Ali, Dalia Benetiene, Komlan Tevi, Hanne Andersen, Mark G. Lewis, Aaron G. Schmidt, Douglas A. Lauffenburger, Galit Alter, Jacob D. Estes, Hanneke Schuitemaker, Roland Zahn, Dan H. Barouch

**Affiliations:** 1 Center for Virology and Vaccine Research, Beth Israel Deaconess Medical Center, Harvard Medical School, Boston, MA USA; 2 Janssen Vaccines & Prevention BV, Leiden, Netherlands; 3 Tufts University Cummings School of Veterinary Medicine, North Grafton, MA USA; 4 Ragon Institute of MGH, MIT, and Harvard, Cambridge, MA USA; 5 Massachusetts Institute of Technology, Cambridge, MA USA; 6 Oregon Health & Sciences University, Beaverton, OR USA; 7 Harvard Medical School, Boston, MA USA; 8 Bioqual, Rockville, MD USA; 9 Massachusetts Consortium on Pathogen Readiness, Boston, MA USA

**Keywords:** SARS-CoV-2, Vaccines

## Abstract

Coronavirus disease 2019 (COVID-19) in humans is often a clinically mild illness, but some individuals develop severe pneumonia, respiratory failure and death^1–4^. Studies of severe acute respiratory syndrome coronavirus 2 (SARS-CoV-2) infection in hamsters^5–7^ and nonhuman primates^8–10^ have generally reported mild clinical disease, and preclinical SARS-CoV-2 vaccine studies have demonstrated reduction of viral replication in the upper and lower respiratory tracts in nonhuman primates^11–13^. Here we show that high-dose intranasal SARS-CoV-2 infection in hamsters results in severe clinical disease, including high levels of virus replication in tissues, extensive pneumonia, weight loss and mortality in a subset of animals. A single immunization with an adenovirus serotype 26 vector-based vaccine expressing a stabilized SARS-CoV-2 spike protein elicited binding and neutralizing antibody responses and protected against SARS-CoV-2-induced weight loss, pneumonia and mortality. These data demonstrate vaccine protection against SARS-CoV-2 clinical disease. This model should prove useful for preclinical studies of SARS-CoV-2 vaccines, therapeutics and pathogenesis.

## Abstract (web summary)

A single immunization with an adenovirus vector-based vaccine expressing a stabilized SARS-CoV-2 spike protein induces protection against SARS-CoV-2-induced weight loss, pneumonia and mortality in hamsters.

## Main

SARS-CoV-2 can infect nonhuman primates^8–10^, hamsters^5–7^, ferrets^14–16^, hACE2 transgenic mice^17,18^ and other species^16^, but clinical disease in these models has generally been mild. A severe pneumonia model would be useful for preclinical evaluation of SARS-CoV-2 vaccines and other countermeasures, because SARS-CoV-2 infection in humans can lead to severe clinical disease, respiratory failure and mortality^1–4^. We assessed the clinical and virologic characteristics of high-dose SARS-CoV-2 infection in hamsters and evaluated the protective efficacy of an adenovirus serotype 26 (Ad26) vector-based vaccine^19^ encoding a stabilized SARS-CoV-2 spike (S) in this stringent model.

**Fig. 1:**
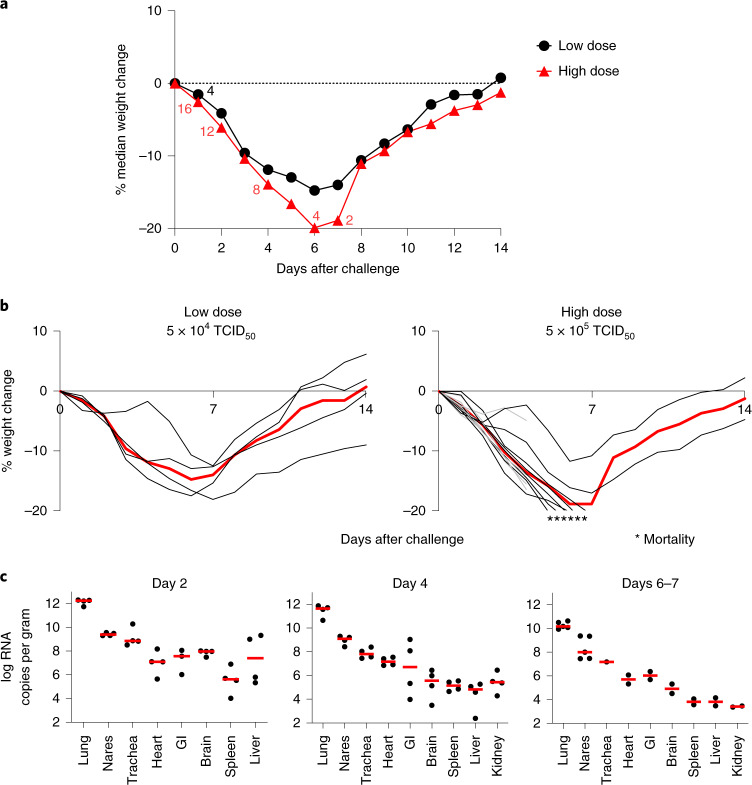
Clinical disease after SARS-CoV-2 infection in hamsters. Syrian golden hamsters (10–12 weeks old; male and female; *n* = 20) were infected with 5 × 10^4^ TCID_50_ (low-dose; *n* = 4) or 5 × 10^5^ TCID_50_ (high-dose; *n* = 16) of SARS-CoV-2 by the intranasal route. **a**, Median percent weight change after challenge. The numbers reflect the number of animals at each time point. In the high-dose group, four animals were necropsied on day 2, four animals were necropsied on day 4, four animals met euthanization criteria on day 6 and two animals met euthanization criteria on day 7. **b**, Percent weight change after challenge in individual animals. Median weight loss is depicted in red. Asterisks indicate mortality. Gray lines indicate animals with scheduled necropsies on day 2 and day 4. **c**, Tissue viral loads as measured by log_10_ RNA copies per gram of tissue (limit of quantification, 100 copies per gram) in the scheduled necropsies at day 2 and day 4 and in 2–5 of 6 animals that met euthanization criteria on days 6–7. Extended tissues were not harvested on day 6.

We inoculated 20 Syrian golden hamsters (10–12 weeks old) with 5 × 10^4^ 50% tissue culture infective dose (TCID_50_) (*n* = 4; low-dose) or 5 × 10^5^ TCID_50_ (*n* = 16; high-dose) SARS-CoV-2 by the intranasal route. In the high-dose group, four animals were necropsied on day 2, four animals were necropsied on day 4 for tissue viral loads and histopathology and the remaining eight animals were followed longitudinally. All remaining animals were necropsied on day 14. In the low-dose group, hamsters lost a median of 14.7% of body weight by day 6 but fully recovered by day 14 (Fig. 1a,b), consistent with previous studies^5–7^. In the high-dose group, hamsters lost a median of 19.9% of body weight by day 6. Of the eight animals in this group that were followed longitudinally, four met Institutional Animal Care and Use Committee humane euthanasia criteria of more than 20% weight loss and respiratory distress on day 6, and two additional animals met these criteria on day 7. The remaining two animals recovered by day 14. These data demonstrate that high-dose SARS-CoV-2 infection in hamsters led to severe weight loss and partial mortality.

Tissue viral loads were assessed in the four animals that received high-dose SARS-CoV-2 and were necropsied on day 2, the four animals that were necropsied on day 4 and five of six of the animals that met euthanasia criteria on days 6–7 (Fig. 1c). High median tissue viral loads on day 2 of 10^12^ RNA copies per gram in lung tissue and 10^8^–10^9^ RNA copies per gram in nares and trachea were observed, with a median of 10^5^–10^8^ RNA copies per gram in heart, gastrointestinal tract, brain, spleen, liver and kidney, indicative of disseminated infection. By days 6–7, tissue viral loads were approximately 2 logs lower, despite continued weight loss.

**Fig. 2:**
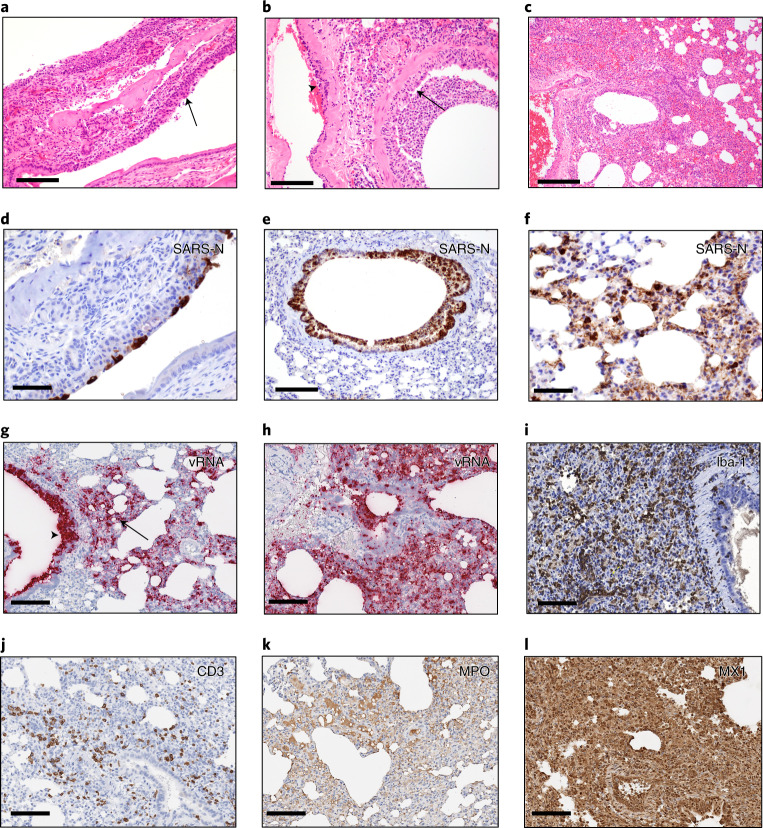
Pathologic features of high-dose SARS-CoV-2 infection in hamsters. **a**, Necrosis and inflammation (arrow) in nasal turbinate, H&E (day 2). **b**, Bronchiolar epithelial necrosis with cellular debris and degenerative neutrophils in lumen (arrow) and transmigration of inflammatory cells in vessel wall (arrowhead), H&E (day 2). **c**, Interstitial pneumonia, hemorrhage and consolidation of lung parenchyma, H&E (day 2). **d**, Nasal turbinate epithelium shows strong positivity for SARS-CoV-N by IHC (day 2). **e**, Bronchiolar epithelium and luminal cellular debris show strong positivity for SARS-CoV-N by IHC (day 2). **f**, Pneumocytes and alveolar septa show multifocal strong positivity for SARS-CoV-N by IHC (day 2). **g**, Diffuse vRNA staining by RNAscope within pulmonary interstitium (arrow, interstitial pneumonia) and within bronchiolar epithelium (arrowhead; day 2). **h**, Diffuse vRNA staining by RNAscope within pulmonary interstitium (day 4). **i**, Iba-1 IHC (macrophages) within pulmonary interstitium (day 7). **j**, CD3^+^ T lymphocytes within pulmonary interstitium, CD3 IHC (day 4). **k**, MPO IHC indicating presence of interstitial neutrophils (day 7). **l**, Interferon inducible gene MX1 IHC shows strong and diffuse positivity throughout the lung (day 4). Representative sections are shown. Experiments were repeated at least three times with similar results. Scale bars, 20 μm (**b**, **d**); 50 μm (**a**, **e**, **f**); and 100 μm (**c**, **g**–**l**). H&E, hematoxylin and eosin.

Hamsters infected with high-dose SARS-CoV-2 were assessed by histopathology on days 2 (*n* = 4), 4 (*n* = 4), 6–7 (*n* = 6) and 14 (*n* = 2). Infection was associated with marked inflammatory infiltrates and multifocal epithelial necrosis of the nasal turbinate (Fig. 2a) and bronchiolar epithelium, resulting in degenerative neutrophils and cellular debris in the lumen (Fig. 2b). The endothelium of nearby vessels was reactive with adherence of mononuclear cells to the endothelium and transmigrating within vessel walls, indicative of endothelialitis (Fig. 2b). There was moderate to severe multifocal interstitial pneumonia characterized by pulmonary consolidation affecting 30–60% of the lung parenchyma as early as day 2 after SARS-CoV-2 infection (Fig. 2c). Inflammatory infiltrates consisted of massive numbers of macrophages and neutrophils with fewer lymphocytes. The nasal turbinate epithelium (Fig. 2d) and bronchiolar epithelial cells (Fig. 2e) were strongly positive for SARS nucleocapsid protein (SARS-CoV-N) by immunohistochemistry (IHC) in regions of inflammation and necrosis. SARS-CoV-N IHC also showed locally extensive staining of the alveolar septa and interstitial mononuclear cells morphologically consistent with macrophages (Fig. 2f). Similarly, substantial SARS-CoV-2 viral RNA (vRNA) was observed in the bronchiolar epithelium and the pulmonary interstitium in regions of inflammation (Fig. 2g,h).

**Extended Data Fig. 1:**
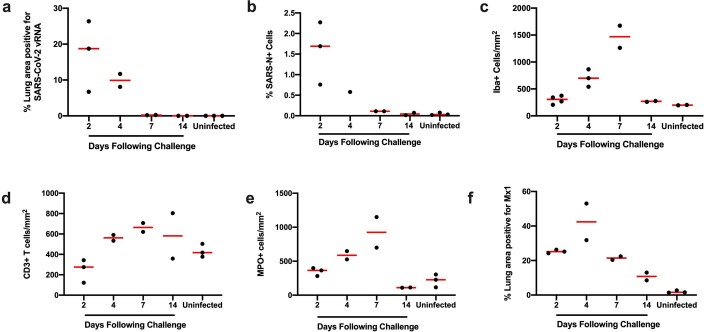
Longitudinal quantitative image analysis of viral replication and associated inflammation in lungs. **a**, Percent lung area positive for anti-sense SARS-CoV-2 viral RNA (vRNA) by RNAscope ISH. **b**, Percentage of total cells positive for SARS-CoV-N protein (nuclear or cytoplasmic) by IHC. **c**, Iba-1 positive cells per unit area by IHC. **d**, CD3 positive cells per unit area. **e**, MPO positive cells per unit area. **f**, Percentage of MX1 positive lung tissue as a proportion of total lung area. ISH, in situ hybridization; IHC, immunohistochemistry; SARS-N, SARS-CoV nucleocapsid; MPO, myeloperoxidase; MX1, myxovirus protein 1 (a type 1 interferon inducible gene). Each dot represents one animal.

**Extended Data Fig. 2:**
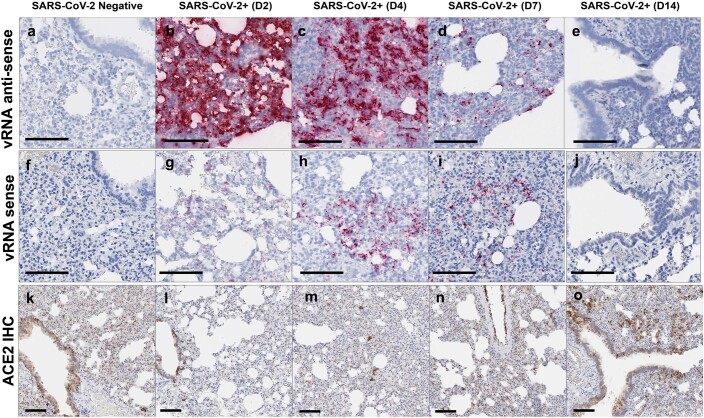
Lung viral dynamics and ACE2 receptor expression patterns. Hamsters were necropsied before (SARS-CoV-2 Negative) or after high-dose SARS-CoV-2 challenge on day 2 (D2), day 4 (D4), day 7 (D7), and day 14 (D14) following challenge. Serial sections of lung tissue were stained for vRNA anti-sense RNAscope **a-e**, for vRNA sense RNAscope **f-j**, and ACE2 IHC **k-o**. Anti-sense RNAscope used a sense probe; sense RNAscope used an anti-sense probe. IHC, immunohistochemistry. Representative sections are shown. Experiments were repeated at least 3 times with similar results. Scale bars = 100 μm.

Levels of both SARS-CoV-2 vRNA and SARS-CoV-N protein expression in lung were highest on day 2 and diminished by day 4, with minimal vRNA and SARS-CoV-N protein detected by day 7 (Extended Data Fig. 1). The pneumonia was characterized by large inflammatory infiltrates of ionized calcium-binding adaptor protein 1 (Iba-1)^+^ macrophages in the lung interstitium as well as CD3^+^ T lymphocytes (Fig. 2i,j). Many viable and degenerative neutrophils were detected throughout the lung, especially in regions of necrosis, with high expression of neutrophil myeloperoxidase (MPO) throughout the lung (Fig. 2k). Diffuse expression of the interferon inducible gene product MX1 was also detected in the lung (Fig. 2l). In contrast with the kinetics of SARS-CoV-2 vRNA and SARS-CoV-N detection, which peaked on day 2, these markers of inflammation peaked on day 7 (Extended Data Fig. 1), coincident with maximal weight loss and mortality (Fig. 1a,b). Detection of vRNA in the lung by RNAscope did not simply reflect the viral inoculum, as we detected not only negative anti-sense vRNA (Extended Data Fig. 2a–e) but also positive-sense vRNA (Extended Data Fig. 2f–j), which overlapped in location and pattern, from day 2 to day 7 after challenge. SARS-CoV-2 vRNA expression (both anti-sense and sense) was present in lung with robust ACE2 receptor expression (Extended Data Fig. 2k–o).

**Extended Data Fig. 3:**
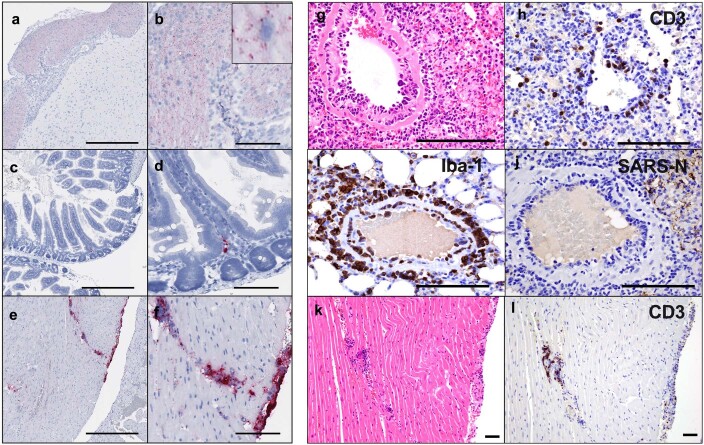
Extrapulmonary pathology. **a**, Anti-sense SARS-CoV-2 viral RNA (vRNA) in brainstem on day 2 following challenge. **b**, Higher magnification showing cytoplasmic vRNA staining in neurons in the absence of inflammation and pathology. **c**, Anti-sense SARS-CoV-2 vRNA staining in the lamina propria of small intestinal villus on day 2 following challenge. **d**, Higher magnification showing cytoplasmic and nuclear vRNA staining in an individual mononuclear cell in the absence of inflammation and tissue pathology. **e**, Anti-sense SARS-CoV-2 vRNA staining within the myocardium and along the epicardial surface of the heart on day 4 following challenge. **f**, Higher magnification showing staining of inflammatory mononuclear cell infiltrates consistent with focal myocarditis. **g**, Pulmonary vessel showing endothelialitis day 4 (d4) following challenge. **h**, Pulmonary vessel showing CD3+ T lymphocyte staining by IHC adhered to endothelium and within vessel wall, d4 following challenge. **i**, Pulmonary vessel showing Iba-1+ staining by IHC of macrophages along endothelium and perivascularly, d4. **j**, Pulmonary vessel showing minimal vascular staining for SARS-CoV-N by IHC, d4. **k**, Heart from (**e**, **f**) showing focal lymphocytic myocarditis as confirmed by CD3+ T lymphocyte staining **l**, of cells by IHC, d4. Representative sections are shown. Experiments were repeated at least 3 times with similar results. Scale bars = 500 μm (**a, c, e**); 100 μm (**b, d, f, g-l**).

**Extended Data Fig. 4:**
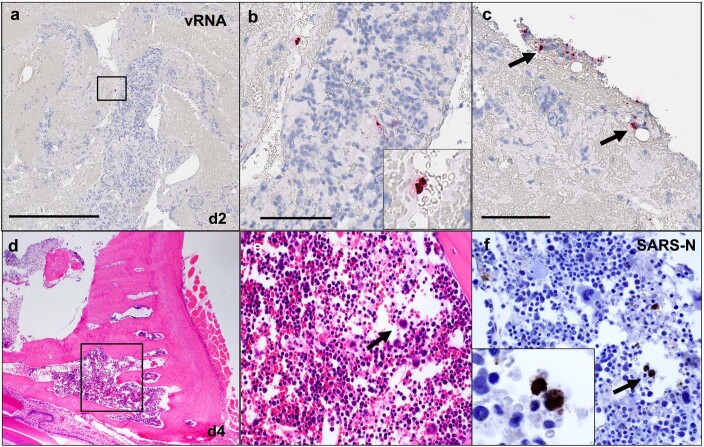
SARS-CoV-2 in blood mononuclear cells and bone marrow. **a-c**, SARS-CoV-2 anti-sense vRNA staining within mononuclear cells within lung thrombus on day 2 following challenge. **d**, Bone marrow from the nasal turbinate 4 days following challenge showing **e**, hematopoetic cells (H&E) that show **f**, positive staining for SARS-CoV-N IHC. vRNA, viral RNA; H&E, hematoxylin and eosin; IHC, immunohistochemistry. Representative sections are shown. Experiments were repeated at least 3 times with similar results. Scale bars = 500 μm (**a**); 200 μm (**d**); 100 μm (**b, c, e, f**).

Systemic vRNA was also detected in distal tissues, including the brain stem, gastrointestinal tract and myocardium (Extended Data Fig. 3a–f). Prominent endothelialitis and perivascular inflammation with macrophages and lymphocytes was observed in these tissues, despite minimal SARS-CoV-N staining (Extended Data Fig. 3g–j). Focal lymphocytic myocarditis was noted in one animal and corresponded to the presence of vRNA (Extended Data Fig. 3k–l). Other sites of virus detection included peripheral blood mononuclear cells in thrombi in lung (Extended Data Fig. 4a–c) and bone marrow of the nasal turbinate (Extended Data Fig. 4d–f).

**Fig. 3:**
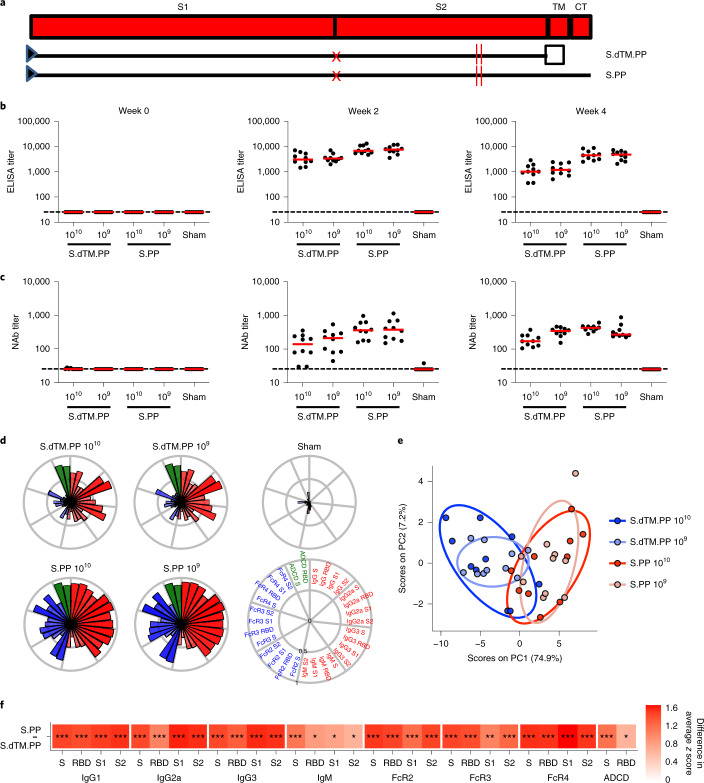
Humoral immune responses in vaccinated hamsters. **a**, SARS-CoV-2 S immunogens with 1) deletion of the transmembrane region and cytoplasmic tail reflecting the soluble ectodomain with a foldon trimerization domain (S.dTM.PP) or 2) full-length S (S.PP), both with mutation of the furin cleavage site and two proline stabilizing mutations. The red X depicts furin cleavage site mutation; red vertical lines depict proline mutations; and the open square depicts the foldon trimerization domain. S1 and S2 represent the first and second domain of the S protein; TM depicts the transmembrane region; and CT depicts the cytoplasmic domain. Hamsters were vaccinated with 10^10^ vp or 10^9^ vp of Ad26-S.dTM.PP or Ad26-S.PP or sham controls (*n* = 10 per group). Humoral immune responses were assessed at weeks 0, 2 and 4 by **b**) RBD-specific binding antibody ELISA and **c**) pseudovirus neutralization assays. Red bars reflect median responses. Dotted lines reflect assay limit of quantitation. **d**, S- and RBD-specific IgG subclass, FcγR and ADCD responses at week 4 are shown as radar plots. The size and color intensity of the wedges indicate the median of the feature for the corresponding group (antibody subclass, red; FcγR binding, blue; ADCD, green). **e**, PCA plot showing the multivariate antibody profiles across vaccination groups. Each dot represents an animal; the color of the dot denotes the group; and the ellipses show the distribution of the groups as 70% confidence levels assuming a multivariate normal distribution. **f**, The heat map shows the differences in the means of *z*-scored features between vaccine groups S.PP and S.dTM.PP. The two groups were compared by two-sided Mann–Whitney tests, and stars indicate the Benjamini–Hochberg-corrected *q* values (*q < 0.05, **q < 0.01 and ***q < 0.001).

We produced recombinant, replication-incompetent Ad26 vectors encoding 1) SARS-CoV-2 S with deletion of the transmembrane region and cytoplasmic tail reflecting the soluble ectodomain with a foldon trimerization domain (S.dTM.PP) or 2) full-length S (S.PP), both with mutation of the furin cleavage site and two proline stabilizing mutations^20^ (Fig. 3a). We recently reported the immunogenicity and protective efficacy of these vaccines against SARS-CoV-2 challenge in rhesus macaques^13^.

**Extended Data Fig. 5:**
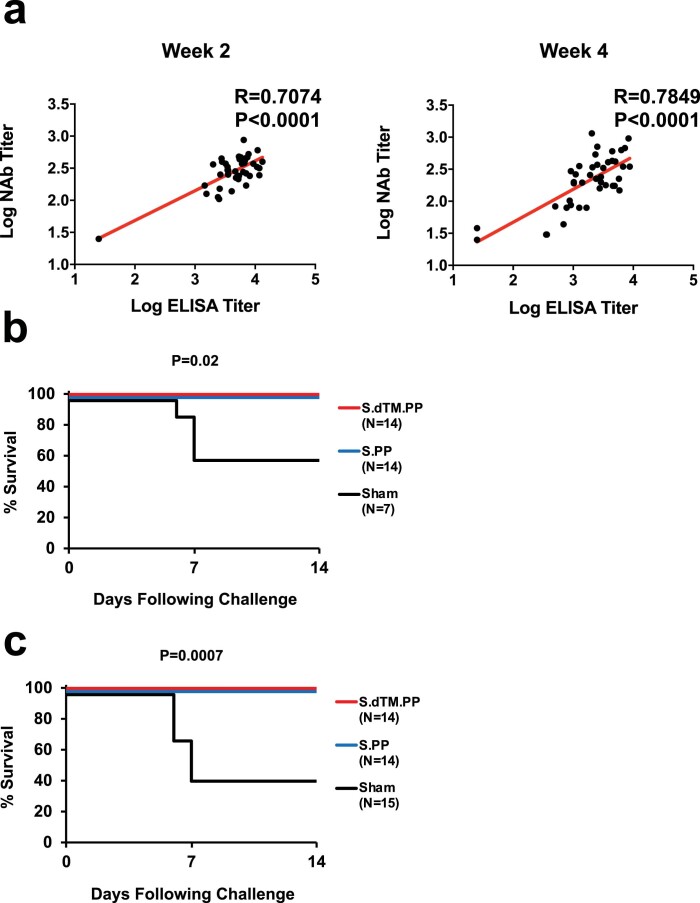
Correlation of antibody titers and survival curves. **a**, Correlations of binding ELISA titers and pseudovirus NAb titers at week 2 and week 4. Red lines reflect the best linear fit relationship between these variables. P and R values reflect two-sided Spearman rank-correlation tests. **b**, Survival curve for the vaccine study. P values indicate two-sided Fisher’s exact tests. N denotes number of animals in each group. **c**, Combined analysis of the two hamster studies involving all animals that received the 5x10^5^ TCID_50_ challenge dose and were followed longitudinally. P values indicate two-sided Fisher’s exact tests. N denotes number of animals in each group.

We immunized 50 Syrian golden hamsters with 10^10^ or 10^9^ viral particles (vp) of Ad26 vectors encoding S.dTM.PP or S.PP (*n* = 10 per group) or sham controls (*n* = 10). Animals received a single vaccination by the intramuscular route at week 0. We observed receptor-binding domain (RBD)-specific binding antibodies by enzyme-linked immunosorbent assay (ELISA)^10,11^ (Fig. 3b) and neutralizing antibodies (NAbs) by a pseudovirus neutralization assay^10,11,21^ (Fig. 3c) in all animals at week 2 and week 4. At week 4, Ad26-S.PP elicited 4.0–4.7-fold higher median ELISA titers (4,470, 4,757) compared to Ad26-S.dTM.PP (1,014, 1,185) (Fig. 3b; *P* < 0.0001, two-sided Mann–Whitney tests). Similarly, Ad26-S.PP elicited 1.8–2.6-fold higher median NAb IC_50_ titers (359, 375) compared to Ad26-S.dTM.PP (139, 211) (*P* < 0.05, two-sided Mann–Whitney tests). For each vector, the two doses tested appeared to be similar. ELISA and NAb data were correlated at both week 2 and week 4 (*R* = 0.7074, *P* < 0.0001 and *R* = 0.7849, *P* < 0.0001, respectively, two-sided Spearmanʼs rank correlation tests; Extended Data Fig. 5a).

We further characterized S-specific and RBD-specific antibody responses in the vaccinated animals at week 4 by systems serology^22^. IgG, IgG2a, IgG3, IgM, Fc-receptors FcRγ2, FcRγ3 and FcRγ4 and antibody-dependent complement deposition (ADCD) responses were assessed (Fig. 3d–f). Higher and more consistent responses were observed with Ad26-S.PP than with Ad26.S.dTM.PP (Fig. 3d,f), and a principal component analysis (PCA) of these antibody features confirmed that these two vaccines had distinct profiles (Fig. 3e).

**Fig. 4:**
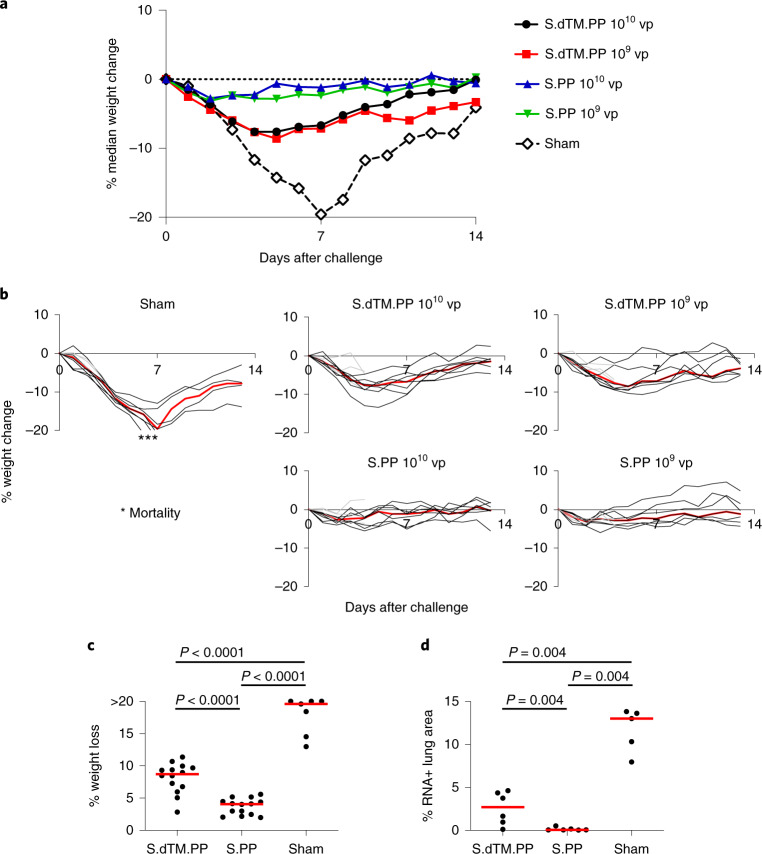
Clinical disease in hamsters after high-dose SARS-CoV-2 challenge. **a**, Median percent weight change after challenge. **b**, Percent weight change after challenge in individual animals. Median weight loss is depicted in red. Asterisks indicate mortality. Gray lines indicate animals with scheduled necropsies on day 4. **c**, Maximal weight loss in the combined Ad26-S.dTM.PP (*n* = 14), Ad26-S.PP (*n* = 14) and sham control (*n* = 7) groups, excluding the animals that were necropsied on day 4. *P* values indicate two-sided Mann–Whitney tests. *n* reflects all animals that were followed for weight loss and were not necropsied on day 4. **d**, Quantification of percent lung area positive for anti-sense vRNA in tissue sections from Ad26-S.dTM.PP and Ad26-S.PP vaccinated hamsters as compared to control hamsters on day 4 after challenge. *P* values represent two-sided Mann–Whitney tests.

**Extended Data Fig. 6:**
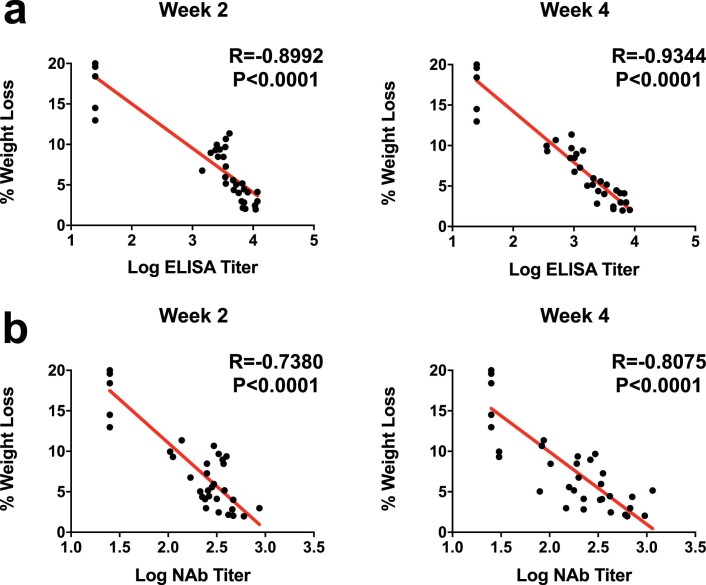
Antibody correlates of clinical protection. Correlations of **a**, binding ELISA titers and **b**, pseudovirus NAb titers at week 2 and week 4 with maximum percent weight loss following challenge. Red lines reflect the best linear fit relationship between these variables. P and R values reflect two-sided Spearman rank-correlation tests.

At week 4, all animals were challenged with 5 × 10^5^ TCID_50_ SARS-CoV-2 by the intranasal route. Three animals in each group were necropsied on day 4 for tissue viral loads and histopathology, and the remaining seven animals in each group were followed until day 14. In the sham controls, hamsters lost a median of 19.6% of body weight by day 7, and 43% (3/7) of the animals that were followed longitudinally met euthanasia criteria on days 6–7 (Fig. 4a,b). The Ad26-S.dTM.PP vaccinated animals lost a median of 8.7% body weight, and the Ad26-S.PP vaccinated animals lost a median of 4.0% body weight (Fig. 4a,b). Maximum percent weight loss was markedly lower in both vaccinated groups compared to sham controls (*P* < 0.0001, two-sided Mann–Whitney tests; Fig. 4c), and animals that received Ad26-S.PP showed less weight loss than animals that received Ad26.S.dTM.PP (*P* < 0.0001, two-sided Mann–Whitney tests; Fig. 4c). Both vaccines protected against mortality, defined as meeting humane euthanization criteria, as compared to sham controls (*P* = 0.02, two-sided Fisher’s exact tests; Extended Data Fig. 5b). A combined analysis of the two hamster experiments confirmed that both vaccines effectively protected against mortality (*P* = 0.007, two-sided Fisher’s exact tests; Extended Data Fig. 5c). ELISA responses at week 2 (*R* = −0.8992, *P* < 0.0001) and week 4 (*R* = −0.9344, *P* < 0.0001) correlated inversely with maximum percent weight loss (Extended Data Fig. 6a). NAb responses at week 2 (*R* = −0.7380, *P* < 0.0001) and week 4 (*R* = −0.8075, *P* < 0.0001) also correlated inversely with maximum percent weight loss (Extended Data Fig. 6b).

**Extended Data Fig. 7:**
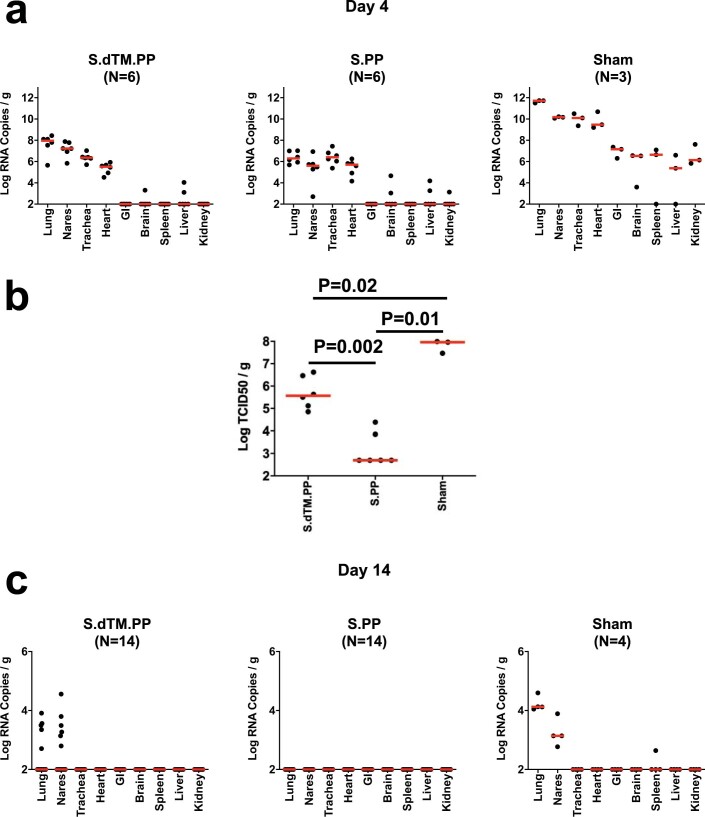
Tissue viral loads on day 4 and day 14. Tissue viral loads as measured by **a**, log_10_ subgenomic RNA copies per gram tissue (limit of quantification 100 copies/g) and **b**, log_10_ infectious virus TCID50 titers per gram tissue (limit of quantification 100 TCID50/g) on day 4 (N=6 reflects both dose groups for each vaccine) and **c**, log_10_ subgenomic RNA copies per gram tissue on day 14 (N=14 reflects both dose groups for each vaccine) following challenge. Red lines reflect median values. Each dot represents one animal.

Tissue viral loads were assessed in the subset of animals necropsied on day 4 and in the remaining surviving animals on day 14. On day 4 after high-dose SARS-CoV-2 challenge, virus was detected in tissues in all animals by subgenomic RNA reverse trancription–polymerase chain reaction (RT–PCR), which is thought to measure replicating virus^10,23^ (Extended Data Fig. 7a). Median viral loads in lung tissue were approximately 10^12^ RNA copies per gram in the sham controls compared to 10^8^ RNA copies per gram in the Ad26-S.dTM.PP vaccinated animals and 10^6^ RNA copies per gram in the Ad26-S.PP vaccinated animals. Reduced TCID_50_ infectious virus titers per gram of lung tissue were also observed for the Ad26-S.dTM.PP and Ad26.S.PP vaccinated animals compared to sham controls (*P* = 0.02 and *P* = 0.01, respectively, two-sided Mann–Whitney tests; Extended Data Fig. 7b). By day 14, virus was still detected in lung and nares of the surviving sham controls but was observed in only a minority of Ad26-S.dTM.PP vaccinated animals and in none of the Ad26-S.PP vaccinated animals (Extended Data Fig. 7c).

**Extended Data Fig. 8:**
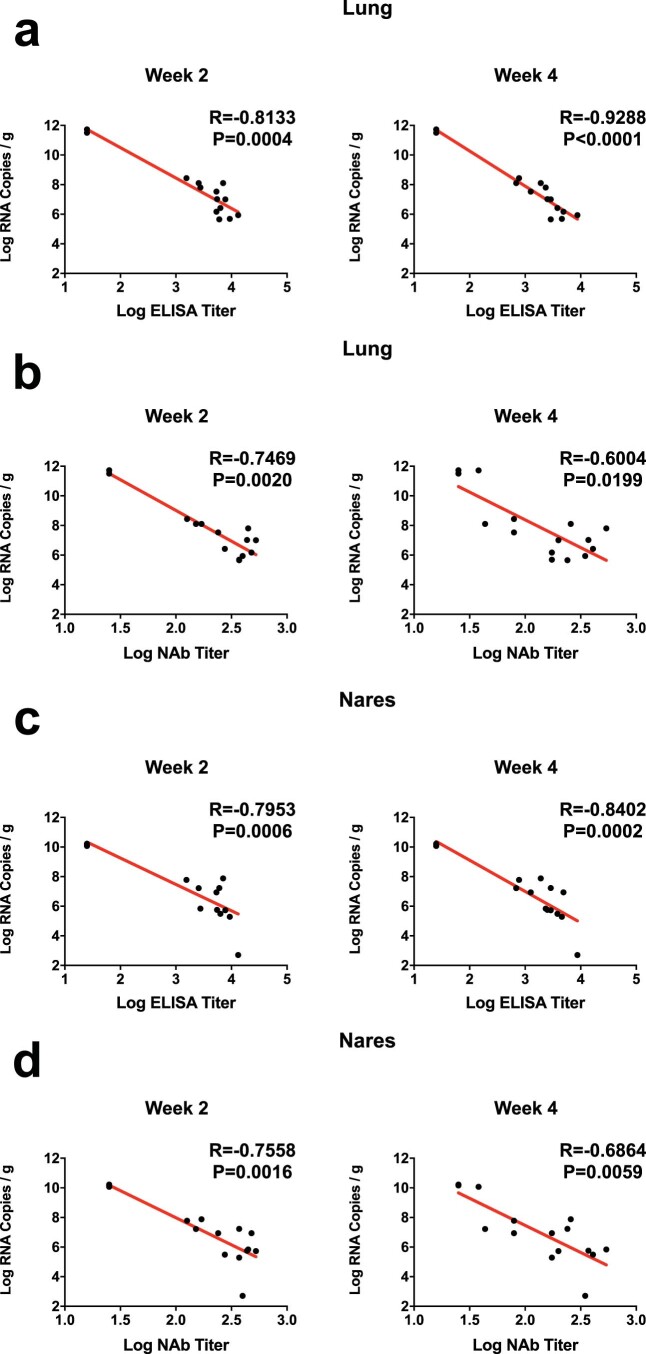
Antibody correlates of protection. Correlations of **a, c**, binding ELISA titers and **b, d**, pseudovirus NAb titers at week 2 and week 4 with log_10_ RNA copies per gram (**a, b**) lung and (**c, d**) nasal turbinate tissue in the animals that were necropsied on day 4. Red lines reflect the best linear fit relationship between these variables. P and R values reflect two-sided Spearman rank-correlation tests.

**Extended Data Fig. 9:**
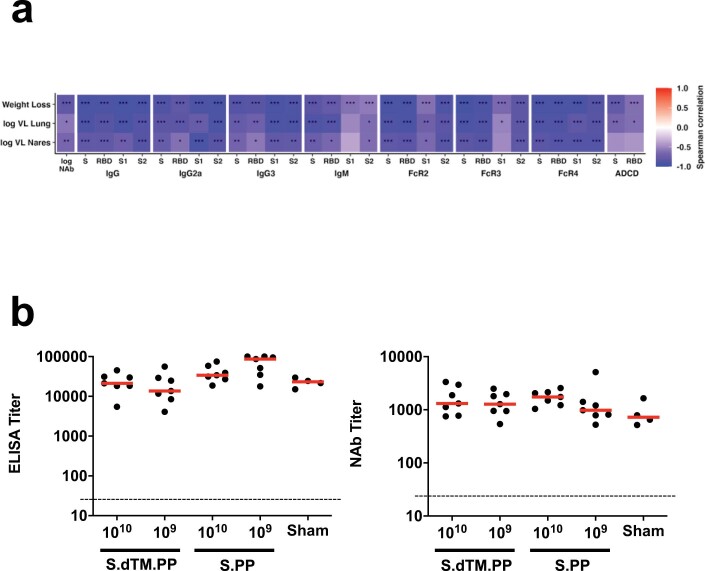
Antibody correlates of protection and anamnestic responses. **a**, The heatmaps show the Spearman rank correlation between antibody features and weight loss (N=35), lung viral loads (N=12), and nasal turbinate viral loads (N=12). N reflects all animals that were followed for weight loss or that were necropsied for lung or nasal turbinate viral loads. Significant correlations are indicated by stars after multiple testing correction using the Benjamini-Hochberg procedure (*q < 0.05, ** q < 0.01, *** q < 0.001). **b**, ELISA and NAb responses in surviving hamsters on day 14 following SARS-CoV-2 challenge.

ELISA responses at week 2 (*R* = −0.8133, *P* = 0.0004) and week 4 (*R* = −0.9288, *P* < 0.0001) correlated inversely with lung viral loads at day 4 (Extended Data Fig. 8a), and NAb responses at week 2 (*R* = −0.7469, *P* = 0.0020) and week 4 (*R* = −0.6004, *P* = 0.0199) correlated inversely with lung viral loads at day 4 (Extended Data Fig. 8b). ELISA and NAb responses also correlated inversely with viral loads in nasal turbinates (Extended Data Fig. 8c,d). A deeper analysis of immune correlates revealed that multiple antibody characteristics correlated inversely with weight loss and tissue viral loads (Extended Data Fig. 9a).

The surviving sham controls developed potent binding and neutralizing antibody responses by day 14 after challenge (Extended Data Fig. 9b). Vaccinated animals also demonstrated higher ELISA and NAb responses after challenge (Extended Data Fig. 9b), consistent with tissue viral loads showing low and transient levels of virus replication in these animals after high-dose SARS-CoV-2 challenge.

**Extended Data Fig. 10:**
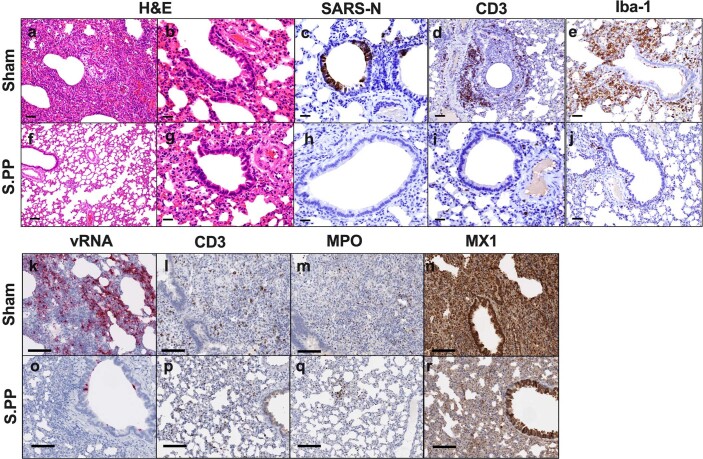
Ad26 vaccination protects against SARS-CoV-2 pathology. Histopathological H&E evaluation of **a–e, k–n**, sham controls and **f–j, o–r**, Ad26-S.PP vaccinated hamsters shows in sham controls (**a**) severe consolidation of lung parenchyma and infiltrates of inflammatory cells, (**b**) bronchiolar epithelial syncytia and necrosis, (**c**) SARS-CoV-N positive (IHC) bronchiolar epithelial cells, (**d**) peribronchiolar CD3+ T lymphocyte infiltrates, and (**e**) peribronchiolar macrophage infiltrates (Iba-1; IHC), and (**f-j**) minimal to no corresponding pathology in Ad26-S.PP vaccinated animals. SARS-CoV-2 anti-sense RNAscope ISH in (**k**), interstitial CD3+ T lymphocytes **l**) MPO staining by IHC, and MX1 staining by IHC **n**) in sham controls compared to similar regions in Ad26-S.PP vaccinated animals **o-r**) on day 4 following challenge. Representative sections are shown. Experiments were repeated at least 3 times with similar results. Scale bars = 20 μm (**b, c, g–i**); 50 μm (**a, d, e, j**); 100 μm (**f, k–r**).

Vaccinated animals also demonstrated diminished pathology compared to sham controls on day 4 after challenge (Extended Data Fig. 10). Ad26-S.PP vaccinated animals demonstrated minimal to no evidence of viral interstitial pneumonia, disruption of the bronchiolar epithelium or peribronchiolar aggregates of CD3^+^ T lymphocytes and macrophages. Histiocytic and neutrophilic inflammatory infiltrates were markedly reduced in all lung lobes, and significantly reduced SARS-CoV-2 vRNA was observed in Ad26-S.dTM.PP and Ad26-S.PP vaccinated hamsters compared to sham controls (*P* = 0.004 and *P* = 0.004, respectively, two-sided Mann–Whitney tests; Fig. 4d).

In this study, we demonstrated that a single immunization of an Ad26 vector encoding a full-length prefusion stabilized S immunogen (S.PP) protected against severe clinical disease after high-dose SARS-CoV-2 challenge in hamsters. Sham controls demonstrated marked weight loss, severe pneumonia and partial mortality. In contrast, vaccinated animals showed minimal weight loss and pneumonia and no mortality. Vaccine-elicited binding and neutralizing antibody responses correlated with protection against clinical disease as well as reduced virus replication in the upper and lower respiratory tract.

The severity of clinical disease in this model contrasts with previous studies involving SARS-CoV-2 infection in hamsters^5–7^ and other species^8–10,14–18^. Hamsters are a permissive model for SARS-CoV-2 as a result of their homology to the human ACE2 receptor^5^, and transmission among hamsters has been reported^6^. The high challenge dose resulted in extensive clinical disease in the present study, although biologic factors that remain to be fully defined might also affect clinical disease, such as animal age, animal origin and viral challenge stock.

SARS-CoV-2 vaccine studies in nonhuman primates have, to date, demonstrated protection against infection or reduction of viral replication in the upper and lower respiratory tracts^11,12^. We have also recently reported that a single immunization of Ad26-S.PP provided complete or near-complete protection against SARS-CoV-2 challenge in rhesus macaques^13^. However, SARS-CoV-2 infection in nonhuman primates does not result in severe clinical disease or mortality^8–10^. A severe disease model would be useful to complement current nonhuman primate challenge models, because protection against viral replication does not necessarily imply protection against severe disease. Indeed, in the histopathologic analysis of hamsters in the present study, viral loads in lung decreased from day 2 to day 7, whereas inflammatory markers continued to escalate during this time period and correlated with continued weight loss. These data suggest that progressive clinical disease in hamsters is primarily an inflammatory process, which is triggered by infection but continued to increase even when viral replication decreased.

Because COVID-19 in humans can progress to severe clinical disease, it is important to test SARS-CoV-2 vaccine candidates in preclinical models that recapitulate severe clinical disease, including fulminant pneumonia and mortality. The high-dose hamster model described here achieves many of these criteria and, therefore, might be useful to study the pathogenesis of severe disease and to test countermeasures. The primary manifestation of clinical disease in this model was severe pneumonia rather than encephalitis that has been reported in certain hACE2 transgenic mouse models^24^. Moreover, binding and neutralizing antibody responses correlated with protection.

In summary, our data demonstrate that a single immunization of Ad26-S.PP provides robust protection against severe clinical disease after high-dose SARS-CoV-2 infection in hamsters. To our knowledge, vaccine protection against severe SARS-CoV-2 pneumonia and mortality has not previously been reported. Ad26-S.PP, which is also termed Ad26.COV2.S, is currently being evaluated in clinical trials. This hamster severe disease model should prove useful for testing of SARS-CoV-2 vaccines, therapeutics and other countermeasures.

## Methods

### Animals and study design

Seventy male and female Syrian golden hamsters (Envigo), 10–12 weeks old, were randomly allocated to groups. All animals were housed at Bioqual. Animals received Ad26 vectors expressing S.dTM.PP or S.PP or sham controls (*n* = 10 per group). Animals received a single immunization of 10^10^ or 10^9^ vp Ad26 vectors by the intramuscular route without adjuvant at week 0. At week 4, all animals were challenged with 5.0 × 10^5^ TCID_50_ (6 × 10^8^ vp, 5.5 × 10^4^ plaque-forming units (PFU)) or 5.0 × 10^4^ TCID_50_ (6 × 10^7^ vp, 5.5 × 10^3^ PFU) SARS-CoV-2, which was derived with one passage from USA-WA1/2020 (NR-52281, BEI Resources)^10^. Virus was administered as 100 μl by the intranasal route (50 μl in each nare). Body weights were assessed daily. All immunologic and virologic assays were performed blinded. On day 4, a subset of animals was euthanized for tissue viral loads and pathology. All animal studies were conducted in compliance with all relevant local, state and federal regulations and were approved by the Bioqual Institutional Animal Care and Use Committee.

### Ad26 vectors

Ad26 vectors were constructed with two variants of the SARS-CoV-2 S protein sequence (Wuhan/WIV04/2019; GenBank MN996528.1). Sequences were codon optimized and synthesized. Replication-incompetent, E1/E3-deleted Ad26-vectors^19^ were produced in PER.C6.TetR cells using a plasmid containing the full Ad26 vector genome and a transgene expression cassette. Sham controls included Ad26-Empty vectors. Vectors were sequenced and tested for expression before use.

### Histopathology and IHC

Tissues were fixed in freshly prepared 4% paraformaldehyde for 24 h, transferred to 70% ethanol, paraffin embedded within 7–10 days and block sectioned at 5 µm. Slides were baked for 30–60 min at 65 ^°^C and then deparaffinized in xylene and rehydrated through a series of graded ethanol to distilled water. For SARS-CoV-N, Iba-1 and CD3 IHC, heat-induced epitope retrieval was performed using a pressure cooker on steam setting for 25 min in citrate buffer (Thermo Fisher Scientific, AP-9003–500), followed by treatment with 3% hydrogen peroxide. Slides were then rinsed in distilled water and protein blocked (Biocare, BE965H) for 15 min followed by rinses in 1× PBS. Primary rabbit anti-SARS-CoV-nucleoprotein antibody (Novus, NB100–56576, at 1:500 or 1:1,000), rabbit anti-Iba-1 antibody (Wako, 019–19741, at 1:500) or rabbit anti-CD3 (Dako, A0452, at 1:300) was applied for 30 min, followed by rabbit Mach-2 HRP-Polymer (Biocare, RHRP520L) for 30 min and then counterstained with hematoxylin followed by bluing using 0.25% ammonia water. Labeling for SARS-CoV-N, Iba-1 and CD3 was performed on a Biogenex i6000 Autostainer (v3.02). In some cases, CD3, Iba-1 and ACE-2 staining was performed with CD3 at 1:400 (Thermo Fisher Scientific, cat. no. RM-9107-S, clone SP7), Iba-1 at 1:500 (Biocare, cat. no. CP290A, polyclonal) or ACE-2 (Abcam, ab108252), all of which were detected by using Rabbit Polink-1 HRP (GBI Labs, cat. no. D13–110). Neutrophil (MPO) and type 1 IFN response (Mx1) was performed with MPO (Dako, cat. no. A0398, polyclonal) at 1:1,000 detection using Rabbit Polink-1 HRP and Mx1 (EMD Millipore, cat. no. MABF938, clone M143/CL143) at 1:1,000 detection using Mouse Polink-2 HRP (GBI Labs, cat. no. D37–110). Staining for CD3, Iba-1, MPO and Mx1 IHC was performed as previously described using a Biocare intelliPATH autostainer, with all antibodies being incubated for 1 h at room temperature. Tissue pathology was assessed independently by two veterinary pathologists (A.J.M. and C.P.M.).

### RNAscope in situ hybridization

RNAscope in situ hybridization was performed as previously described^10^ using SARS-CoV2 anti-sense specific probe v-nCoV2019-S (ACD, cat. no. 848561) targeting the positive-sense viral RNA and SARS-CoV2 sense specific probe v-nCoV2019-orf1ab-sense (ACD, cat. no. 859151) targeting the negative-sense genomic vRNA. In brief, after slides were deparaffinized in xylene and rehydrated through a series of graded ethanol to distilled water, retrieval was performed for 30 min in ACD P2 retrieval buffer (ACD, cat. no. 322000) at 95–98 ^°^C, followed by treatment with protease III (ACD, cat. no. 322337) diluted 1:10 in PBS for 20 min at 40 °C. Slides were then incubated with 3% H_2_O_2_ in PBS for 10 min at room temperature. Before hybridization, probe stocks were centrifuged at 13,000 r.p.m. using a microcentrifuge for 10 min and then diluted 1:2 in probe diluent (ACD, cat. no. 300041) to reduce probe aggregation tissue artifacts. Slides were developed using the RNAscope 2.5 HD Detection Reagents-RED (ACD, cat. no. 322360).

### Quantitative image analysis

Quantitative image analysis was performed using HALO software (v2.3.2089.27 or v3.0.311.405; Indica Labs) on at least one lung lobe cross-section from each animal. In cases where more than one cross-section was available, each lung lobe was quantified as an individual data point. For SARS-CoV-N, the Multiplex IHC v2.3.4 algorithm was used with an exclusion screen for acid hematin to determine the percentage of SAR-N protein positive cells as a proportion of the total number of cells. For Iba-1, the Multiplex IHC v2.3.4 algorithm was used for quantitation. For SARS-CoV-2 RNAscope ISH and Mx1 quantification, the Area Quantification v2.1.3 module was used to determine the percentage of total SARS-CoV-2 anti-sense or sense probe or Mx1 protein as a proportion of the total tissue area. For MPO (neutrophil) and CD3^+^ cell quantification, slides were annotated to exclude blood vessels (>5 mm^2^), bronchi, bronchioles, cartilage and connective tissue; subsequently, the Cytonuclear v1.6 module was used to detect MPO^+^ or CD3^+^ cells and frequency was calculated as a proportion of total alveolar tissue (polymorphonuclear leukocytes per mm^2^), determined by running the Area Quantification v2.1.3 module. In all instances, manual inspection of all images was performed on each sample to ensure that the annotations were accurate.

### Subgenomic mRNA assay

SARS-CoV-2 E gene subgenomic mRNA (sgmRNA) was assessed by RT–PCR using primers and probes as previously described^10,11,23^. Briefly, total RNA was extracted from tissue homogenates from several anatomical sites using a QIAcube HT (Qiagen) and RNeasy 96 QIAcube HT Kit (Qiagen). A standard curve was generated using the SARS-CoV-2 E gene sgmRNA by cloning into a pcDNA3.1 expression plasmid; this insert was transcribed using an AmpliCap-Max T7 High Yield Message Maker Kit (Cellscript). Before RT–PCR, samples collected from challenged animals or standards were reverse transcribed using Superscript III VILO (Invitrogen) according to the manufacturer’s instructions. A Taqman custom gene expression assay (Thermo Fisher Scientific) was designed using the sequences targeting the E gene sgmRNA. Reactions were carried out on QuantStudio 6 and 7 Flex Real-Time PCR Systems (Applied Biosystems) according to the manufacturer’s specifications. Standard curves were used to calculate sgmRNA copies per gram tissue; the quantitative assay sensitivity was 100 copies.

### ELISA

RBD-specific binding antibodies were assessed by ELISA essentially as described^10,11^. Briefly, 96-well plates were coated with 1 µg ml^−1^ of SARS-CoV-2 RBD protein (Aaron Schmidt, Massachusetts Consortium on Pathogen Readiness) or 1 µg ml^−1^ of SARS-CoV-2 S protein (Sino Biological) in 1× Dulbeccoʼs phosphate-buffered saline (DPBS) and incubated at 4 °C overnight. After incubation, plates were washed once with wash buffer (0.05% Tween-20 in 1× DPBS) and blocked with 350 µl of casein block per well for 2–3 h at room temperature. After incubation, the block solution was discarded and plates were blotted dry. Three-fold serial dilutions of heat-inactivated serum in casein block were added to wells, and plates were incubated for 1 h at room temperature. Plates were washed three times and then subsequently incubated for 1 h with 0.1 µg ml^−1^ of anti-hamster IgG HRP (SouthernBiotech) in casein block at room temperature in the dark. Plates were washed three times, and then 100 µl of SeraCare KPL TMB SureBlue Start solution was added to each well; plate development was halted by the addition of 100 µl of SeraCare KPL TMB Stop solution per well. The absorbance at 450 nm was recorded using a VersaMax or Omega microplate reader. ELISA endpoint titers were defined as the highest reciprocal serum dilution that yielded an absorbance two-fold above background.

### Pseudovirus neutralization assay

The SARS-CoV-2 pseudoviruses expressing a luciferase reporter gene were generated in an approach similar to as described previously^10,11,21^. Briefly, the packaging construct psPAX2 (AIDS Resource and Reagent Program), luciferase reporter plasmid pLenti-CMV Puro-Luc (Addgene) and S protein expressing pcDNA3.1-SARS CoV-2 SΔCT were co-transfected into HEK293T cells by lipofectamine 2000 (Thermo Fisher Scientific). The supernatants containing the pseudotype viruses were collected 48 h after transfection; pseudotype viruses were purified by filtration with a 0.45-µm filter. To determine the neutralization activity of the antisera from vaccinated animals, HEK293T-hACE2 cells were seeded in 96-well tissue culture plates at a density of 1.75 × 10^4^ cells per well overnight. Three-fold serial dilutions of heat-inactivated serum samples were prepared and mixed with 50 µl of pseudovirus. The mixture was incubated at 37 °C for 1 h before adding to HEK293T-hACE2 cells. Forty-eight hours after infection, cells were lysed in Steady-Glo Luciferase Assay (Promega) according to the manufacturer’s instructions. SARS-CoV-2 neutralization titers were defined as the sample dilution at which a 50% reduction in relative light units was observed relative to the average of the virus control wells.

### Luminex

To detect relative quantity of antigen-specific antibody titers, a customized Luminex assay was performed as previously described^25^. Hereby, fluorescently labeled microspheres (Luminex) were coupled with SARS-CoV-2 antigens including S protein (Eric Fischer, Dana Farber Cancer Institute), S1 and S2 (Sino Biological), as well as RBD (Aaron Schmidt, Ragon Institute) via covalent N-hydroxysuccinimide (NHS)–ester linkages via EDC (Thermo Fisher Scientific) and Sulfo-NHS (Thermo Fisher Scientific). Then, 1.2 × 10^3^ beads per region and antigen were added to a 384-well plate (Greiner) and incubated with diluted serum (1:90 for IgG2a, IgG3, IgM; 1:500 for total IgG and Fc-receptor binding assays) for 16 h shaking at 900 r.p.m. at 4 °C. After formation of immune complexes, microspheres were washed three times in 0.1% bovine serum albumin and 0.05% Tween-20 (Luminex assay buffer) using an automated plate washer (Tecan). PE-labeled goat anti-mouse IgG, IgG2a, IgG3 and IgM detection antibodies (SouthernBiotech) were diluted in Luminex assay buffer to 0.65 µg ml^−1^ and incubated with beads for 1 h at room temperature while shaking at 900 r.p.m. Similarly, for the Fc-receptor binding profiles, recombinant mouse FcγR2, FcγR3 and FcγR4 (Duke Protein Production Facility) were biotinylated (Thermo Fisher Scientific) and conjugated to Streptavidin-PE for 10 min before addition to samples (SouthernBiotech). These mouse antibodies and proteins are cross-reactive to hamster. The coated beads were then washed and read on a flow cytometer, iQue (IntelliCyt), with a robot arm attached (PAA). Events were gated on each bead region; median fluorescence of PE of bead-positive events was reported. Samples were run in duplicate per each secondary detection agent.

### ADCD

ADCD assays were performed as previously described^26^. Briefly, SARS-CoV-2 S and RBD were biotinylated (Thermo Fisher Scientific) and coupled to 1 μm red fluorescent neutravidin beads (Thermo Fisher Scientific) for 2 h at 37 °C, and excess antigen was washed away afterwards. For the formation of immune complexes, 1.82 × 10^8^ antigen-coated beads were added to each well of a 96-well round bottom plate and incubated with 1:10 diluted samples at 37 °C for 2 h. Lyophilized guinea pig complement was reconstituted according to the manufacturer’s instructions (Cedarlane) with water, and 4 μl per well was added in gelatin veronal buffer containing Mg^2+^ and Ca^2+^ (GVB^++^, Boston BioProducts) to the immune complexes for 20 min at 37 °C. Immune complexes were washed with 15 mM ethylenediaminetetraacetic acid in PBS, and fluorescein-conjugated goat IgG fraction to guinea pig complement C3 (MP Biomedicals) was added. After staining, samples were fixed with 4% paraformaldehyde, and sample acquisition was performed via flow cytometry (IntelliCyt, iQue Screener Plus) using a robot arm (PAA). All events were gated on single cells and bead-positive events; the median of C3-positive events is reported. All samples were run in duplicate on separate days.

### Statistical analysis

Analysis of immunologic, virologic and body weight data was performed using GraphPad Prism 8.4.2 (GraphPad Software). Comparison of data between groups was performed using two-sided Mann–Whitney tests. Mortality was assessed by two-sided Fisher’s exact tests. Correlations were assessed by two-sided Spearmanʼs rank correlation tests. *P* values of less than 0.05 were considered significant. All systems serology data were log_10_ transformed. For the radar plots, each antibody feature was normalized such that its minimal value is 0 and the maximal value is 1 across groups before using the median within a group. A PCA was constructed using the R version 3.6.1 package ‘ropls’ to compare multivariate profiles. For the visualization in the heat map, the differences in the means of the S.dTM.PP and S.PP groups of *z*-scored features were shown. To indicate significances in the heat maps, a Benjamini–Hochberg correction was used to correct for multiple comparisons within a row.

### Reporting Summary

Further information on research design is available in the Nature Research Reporting Summary linked to this article.

## Online content

Any methods, additional references, Nature Research reporting summaries, source data, extended data, supplementary information, acknowledgements, peer review information; details of author contributions and competing interests; and statements of data and code availability are available at 10.1038/s41591-020-1070-6.

## Supplementary information

Reporting Summary

## Data Availability

All data are available in the manuscript or the supplementary material. Correspondence and requests for materials should be addressed to D.H.B. (dbarouch@bidmc.harvard.edu).
