# Supplementary material for: Ad26 vaccine protects against SARS-CoV-2 severe clinical disease in hamsters
Source: Nat Med. 2020 Sep 3;26(11):1694–700. doi: 10.1038/s41591-020-1070-6 (PMC7671939; doi:10.1038/s41591-020-1070-6)
Supplement: Supplementary file 1 — Reporting Summary [file 41591_2020_1070_MOESM1_ESM.pdf]

nature research

Corresponding author(s): Dan Barouch

Reporting Summary

Nature Research wishes to improve the reproducibility of the work that we publish. This form provides structure for consistency and transparency in reporting. For further information on Nature Research policies, see Authors & Referees and the Editorial Policy Checklist.

Statistical parameters

When statistical analyses are reported, confirm that the following items are present in the relevant location (e.g. figure legend, table legend, main text, or Methods section).

n/a	Confirmed	
☐	☒	The exact sample size (n) for each experimental group/condition, given as a discrete number and unit of measurement
☐	☒	An indication of whether measurements were taken from distinct samples or whether the same sample was measured repeatedly
☐	☒	The statistical test(s) used AND whether they are one- or two-sided<br>*Only common tests should be described solely by name; describe more complex techniques in the Methods section.*
☐	☒	A description of all covariates tested
☐	☒	A description of any assumptions or corrections, such as tests of normality and adjustment for multiple comparisons
☐	☒	A full description of the statistics including central tendency (e.g. means) or other basic estimates (e.g. regression coefficient) AND variation (e.g. standard deviation) or associated estimates of uncertainty (e.g. confidence intervals)
☐	☒	For null hypothesis testing, the test statistic (e.g. F, t, r) with confidence intervals, effect sizes, degrees of freedom and P value noted<br>*Give P values as exact values whenever suitable.*
☒	☐	For Bayesian analysis, information on the choice of priors and Markov chain Monte Carlo settings
☒	☐	For hierarchical and complex designs, identification of the appropriate level for tests and full reporting of outcomes
☐	☒	Estimates of effect sizes (e.g. Cohen's d, Pearson's r), indicating how they were calculated
☐	☒	Clearly defined error bars<br>*State explicitly what error bars represent (e.g. SD, SE, CI)*

Our web collection on statistics for biologists may be useful.

Software and code

Policy information about availability of computer code

Data collection	No software was used to collect data.
Data analysis	Analysis of virologic and immunologic data was performed using R 3.6.1 and GraphPad Prism 8.4.2 (GraphPad Software).

For manuscripts utilizing custom algorithms or software that are central to the research but not yet described in published literature, software must be made available to editors/reviewers upon request. We strongly encourage code deposition in a community repository (e.g. GitHub). See the Nature Research guidelines for submitting code & software for further information.

Data

Policy information about availability of data

All manuscripts must include a data availability statement. This statement should provide the following information, where applicable:

- Accession codes, unique identifiers, or web links for publicly available datasets
- A list of figures that have associated raw data
- A description of any restrictions on data availability

All data are available in the manuscript or the supplementary material. Correspondence and requests for materials should be addressed to D.H.B. (dbarouch@bidmc.harvard.edu).

Field-specific reporting

Please select the best fit for your research. If you are not sure, read the appropriate sections before making your selection.

☒ Life sciences ☐ Behavioural & social sciences ☐ Ecological, evolutionary & environmental sciences

For a reference copy of the document with all sections, see nature.com/authors/policies/ReportingSummary-flat.pdf

Life sciences study design

All studies must disclose on these points even when the disclosure is negative.

Sample size	Sample size includes N=70 animals (N=10 animals/group given our experience with these vaccines in Mercado et al. Nature 2020). Based on our experience with SARS-CoV-2 in hamsters, this sample size provides power to determine differences in tissue viral loads and clinical endpoints such as weight and mortality.
Data exclusions	No data were excluded.
Replication	Virologic and immunologic measures were performed in duplicate. Technical replicates were minimally different. All attempts at replication were successful.
Randomization	Animals were balanced for gender and otherwise randomly allocated to groups.
Blinding	All immunologic and virologic assays were performed blinded.

Reporting for specific materials, systems and methods

Materials & experimental systems

n/a	Involved in the study	
☐	☒	Unique biological materials
☐	☒	Antibodies
☒	☐	Eukaryotic cell lines
☒	☐	Palaeontology
☐	☒	Animals and other organisms
☒	☐	Human research participants

Methods

n/a	Involved in the study	
☒	☐	ChIP-seq
☒	☐	Flow cytometry
☒	☐	MRI-based neuroimaging

Unique biological materials

Policy information about availability of materials

Obtaining unique materials	SARS-CoV-2 virus stocks are available from BEI. Other reagents can be shared with an MTA for academic research.

Antibodies

Antibodies used	Primary rabbit anti-SARS-CoV-nucleoprotein antibody (Novus; NB100-56576 at 1:500 or 1:1000), rabbit anti-Iba-1 antibody (Wako; 019-19741 at 1:500), rabbit anti-CD3 (Dako; A0452 at 1:300), and antibodies against CD3 at 1:400 (Thermo Cat. No. RM-9107-S; clone SP7), Iba-1 at 1:500 (BioCare Cat. No. CP290A; polyclonal), or ACE-2 (Abcam; ab108252).
Validation	all mAbs used according to manufacturer's instructions and website and previously published methods; mAbs were validated and titrated for specificity prior to use

Animals and other organisms

Policy information about studies involving animals; ARRIVE guidelines recommended for reporting animal research

Laboratory animals	70 male and female Syrian golden hamsters (Envigo), 10-12 weeks old
Wild animals	None

Field-collected samples | None
